# Screening for Hepatocellular Carcinoma

**DOI:** 10.4061/2011/363151

**Published:** 2011-10-17

**Authors:** Hock-Foong Lui

**Affiliations:** ^1^Gleneagles Hospital, 6A Napier Road, Singapore 258500; ^2^Singapore General Hospital, Outram Road, Singapore 169608

## Abstract

Hepatocellular carcinoma is one of the most serious complications of chronic liver disease and is the third most lethal cancer worldwide. Symptoms emerge very late in the course of its natural history with an attendant poor outcome. Screening is of paramount importance in a successful strategy to treat hepatocellular carcinoma. A successful screening program rests the availability of an at-risk population, reliable diagnostics tests that are able to diagnose a condition at a stage where effective, and relatively simple and acceptable treatments are available. In hepatocellular carcinoma, all patients with liver cirrhosis or chronic hepatitis B virus infection are at risk. Six monthly ultrasound and alpha-foetoprotein determination form the backbone of the screening program. Newer modalities and tests show promise but have not supplanted the standard tests.

## 1. Introduction

One of the most serious complications of chronic liver disease is hepatocellular carcinoma. Across the world, it is the 4th most common cancer (age standardised rate of 16 per 100,000) and the 3rd most common cause (age standardised rate of 14.6 per 100,000) of deaths from all cancers, accounting for 700,000 deaths per annum [1]. Although a global cancer, it is especially prevalent in the Asia Pacific and sub-Saharan Africa.

The outcome of a patient after the discovery of hepatocellular carcinoma, like many malignancies, very much depends on the stage of the disease at the time of diagnosis. Curative treatment can be offered to 30% of cases if diagnosed at BCLC stage 0 or A and carries a 5-year survival of 40% to 70%. If diagnosed at stages B or C, the median survival with treatment is 11–20 months. At stage D, only symptomatic treatment is possible and survival does not exceed 3 months [2]. Unfortunately, the lack of symptoms for most part of the natural history of hepatocellular carcinoma is such that many cases are discovered at late stages, limiting the survival of these cases. It is the leading cause of mortality in patients with compensated liver cirrhosis [3]. 

One of the main strategies for prolonging survival in patients with chronic liver disease therefore lies in the diagnosing hepatocellular carcinoma in the early stages so that effective therapy can be offered. In this regard, screening for hepatocellular carcinoma, that is, detection before the onset of symptoms, forms the backbone of such a strategy.

## 2. Screening for Diseases

Screening refers to the detection of a condition whilst it is still without sign or symptom. The repeated application of screening is termed surveillance. The primary aim of screening is to pick up a disease at a stage where treatment is more effective and the outcome, usually measured as survival, is better compared with a later stage of discovery of the condition. The World Health Organisation in 1968 published the following desirable criteria for a condition to be screened [4].

1. The condition should be an important health problem.
2. There should be treatment for the condition.
3. Facilities for diagnosis and treatment should be available.
4. There should be a test or examination for the condition.
5. The test should be acceptable to the population.
6. The natural history of the condition should be adequately understood.
7. There should be an agreed policy on who to treat.
8. The total cost of finding the case should be economically balanced in relation to medical expenditure as a whole.
9. Case finding should be a continuous process, not just a “once and for all” project.

Paraphrasing the above, the ideal screening programme should meet the following criteria:

1. a sufficiently high prevalence of the disease in the population to be screened;
2. a reliable method for screening, that is, it should have a high true positive rate or low false negative rate (high sensitivity) and a high true negative rate or low false positive rate (high specificity);
3. the method of screening should also be easily available, inexpensive, and carries little or no risk of harm to the individual screened;
4. an effective treatment should be available for the disease to positively impact on the survival of the individual.

Currently the hepatocellular carcinoma screening programmes advocated by various expert bodies have a high degree of concurrence. The ensuing discussion will review the merits of hepatocellular carcinoma screening vis-à-vis the desired characteristics of an ideal screening programme as listed above.

## 3. Prevalence of Hepatocellular Carcinoma

Hepatocellular carcinoma develops almost exclusively in the setting of chronic liver disease. The risk of developing hepatocellular carcinoma in this group is over 200 times that of the general population [5]. The risk factors are liver cirrhosis (where the repeated inflammation-necrosis-regeneration cycle increases the risk of carcinogenesis) and chronic hepatitis B virus infection (where the incorporation of hepatitis B virus genome into the hepatocyte DNA increases the risk of carcinogenesis). The risk of hepatocellular carcinoma development varies across these conditions and depends on several factors: age, gender, presence of family history of hepatocellular carcinoma, exposure to environmental factors such as aflatoxins, and aetiology of cirrhosis [6]. Hepatitis C-related cirrhosis appears to be associated with the highest risk of hepatocellular carcinoma, with a 5-year cumulative risk of 17–30%. Haemochromatosis-related cirrhosis carries a 5-year cumulative incidence of 21% whereas hepatitis B-related cirrhosis has a 5-year cumulative incidence of 10–15% depending on endemicity. The corresponding figures for alcoholic cirrhosis and advanced primary biliary cirrhosis are 8% and 4%, respectively [3]. Individuals with chronic hepatitis B virus infections without cirrhosis have a 0.5% annual risk of hepatocellular carcinoma. In this group the risk is higher with advancing age, in men and in those with a family history of hepatocellular carcinoma. Women are at lower risk but the risk is increased in women above the age of 50 years old [7, 8]. Chronic hepatitis B patients with liver cirrhosis have an annual risk of hepatocellular carcinoma of 3–8%. In patients with liver cirrhosis caused by hepatitis C virus infection or advanced primary biliary cirrhosis (stage 4), the annual risk of hepatocellular carcinoma is also high at 3–8%. Cirrhosis caused by other aetiologies such as genetic haemochromatosis and alpha-1-antitrypsin deficiency carry with it a lower annual risk of hepatocellular carcinoma of around 1.5% [9–13]. It is clear that hepatocellular carcinoma can develop from cirrhosis arising from nonalcoholic fatty liver disorder. However the incidence is not clear [14, 15]. 

The effectiveness of a screening strategy can be measured by improvement in survival against the cost incurred to achieve this outcome (cost for each year of life gained). Other outcome measures such as quality of life gained, years of economically viable life gained are important but are more difficult to assess. The thresholds for each of these measures will vary according to cultures, individual outlook, and economic status of a country and are arbitrary. 

Utilising the principles of decision analysis and costeffectiveness, the generally accepted threshold for life gain is 3 months, achieved at a cost of less than US $50,000 per year of life gained. Applying this to decide on the threshold for screening hepatocellular carcinoma, the annual incidence for screening noncirrhotic patients is to be 0.2% and above, and for cirrhotic patients, this translates to an annual risk of 1.5% and above. The difference in the threshold incidences between noncirrhosis and cirrhosis lies in the lower quantum of life gain when hepatocellular carcinoma is diagnosed in an individual with liver cirrhosis.

Based on the above assumptions and thresholds, there is no justification for population-wide screening of hepatocellular carcinoma. Current screening strategies centre on selecting high risk groups for screening [16]. The at-risk groups meeting the above cost-effective criteria for hepatocellular carcinoma screening are

1. patients with liver cirrhosis,
2. male patients with chronic hepatitis B virus infection without cirrhosis who are above the age of 40 years,
3. female patients with chronic hepatitis B virus infection without cirrhosis who are above the age of 50 years,
4. patients with chronic hepatitis B virus infection who have a family history of hepatocellular carcinoma.

For younger individuals with chronic hepatitis B virus infection, the annual risk of hepatocellular carcinoma development is lower than the threshold of 0.2%, but the disease is often more aggressive [17]. Whether the above thresholds should be used to decide if screening should be recommended for this group should be discussed. Whilst not “costeffective”, the discovery of an early tumour in this age group brings about the greatest potential gain in terms of survival and also economically useful years gained, not to mention the less tangible but no less important benefit of averting the trauma for the patient and family of coming to terms with a fatal cancer in a young individual. 

The risk of HCC in patients with chronic HBV infection can also be further stratified using prognostic variables—age, gender, indices of necroinflammation—alanine transaminase level, and HBV DNA load. A prognostic scoring system was developed and validated in a large Asian population with chronic HBV and gives the risk of HCC development at 3, 5, and 10 years [18]. This scoring system has the potential to be applied to refine the decision-making process with respect to screening in less clear-cut situations, for example, in the group discussed above. 

## 4. Reliable Method of Screening

Currently available methods to diagnose hepatocellular carcinoma comprise blood tests to detect elevation of “tumour markers” and imaging modalities. Blood tests that are elevated with hepatocellular carcinoma include AFP (alpha-fetoprotein), the more specific AFP-L3 (L3 subfraction of AFP), and DCP (descarboxy prothrombin) [19–21]. AFP is released by regenerating hepatocytes, malignant hepatocytes, and also extrahepatic sources such as placental cells and germ cells. Among the nonmalignant causes that cause elevated AFP are inflammatory liver conditions, pregnancy, and molar pregnancy. The AFP-L3 is the glycosylated subfraction of AFP and is specific to malignant hepatocytes. It is useful in discriminating between AFP elevation arising from a benign condition against that arising from hepatocellular carcinoma. However its sensitivity is low in cases where the AFP is not markedly elevated. 

DCP is also specific to hepatocellular carcinoma; it is found especially elevated when there is invasion of vascular structures and is a marker of more advanced hepatocellular carcinoma, and therefore may not be a suitable screening tool.

AFP, taken at a level of 20 ng/mL, has a sensitivity of 66% and a specificity of 82% for hepatocellular carcinoma. AFP-L3 has not been studied adequately for screening of hepatocellular carcinoma. Whilst specific, it will likely suffer from decreased sensitivity and is not recommended as a general screening tool. More recently the highly sensitive AFP-L3 (hs-AFP-L3) was evaluated in individuals whose AFP was <20 ng/mL [22]. It was found to increase the sensitivity of detecting HCC from 7% with AFP-L3 to 41.5% with hs-AFP-L3. In addition, it also predicted poorer outcomes in patients with HCC. At present AFP-L3 and hs-AFP-L3 remain adjunctive tools in further evaluation in cases of raised AFP and is not in a position to supplant the use of AFP as a screening tool. As it stands, the present accepted blood test for screening is AFP.

Liver imaging modalities that have proven effective in detecting hepatocellular carcinoma are ultrasound, CT scan, and MRI scan of the liver. In trained operators, ultrasound reliably detects liver nodules above the dimensions of 5 mm. Hepatocellular carcinoma may appear hypoechoic but may be isoechoic or hyperechoic. Other pathological conditions may share similar ultrasonic characteristics. Ultrasound has a sensitivity of 65–80% and a specificity of 90% [16]. It is a test that is generally reproducible, does not carry any adverse effects, and is economical. One limitation of ultrasonography is the difficulty in obtaining a good study in obese patients. 

CT scan of the liver and MRI of the liver provide diagnostic details superior to ultrasound. CT scan is now widely available. Its cost has decreased with economies of scale. However, the radiation exposure is significant, raising the risk of carcinogenesis if used repeatedly [17]. Whilst advocated by some for screening of hepatocellular carcinoma, it is still not yet accepted and therefore not recommended for screening. MRI offers superior resolution. Whilst it does not have the drawback of radiation danger, it is an expensive test, and the acquisition time for one study is considerable, limiting its use as a screening test. At present the use of wither MRI or CT scan of the liver lies in the diagnosis of hepatocellular carcinoma where the screening tests (either ultrasound or AFP) have flagged up suspicion.

At present the combined use of AFP and ultrasound is the recommended mode for screening. The interval of screening should be such that the growth of cancer should be picked up between 2 screening. Too short an interval is a waste of healthcare resources and inconveniences the patient. Too long an interval, in the other hand, runs the risk of allowing the cancer to grow to a later stage, thereby compromising the effectiveness of the whole screening process. The determinant of this interval is the rate of growth or doubling time of the cancer. Studies involving HCV patients suggest that 12-month screening interval brings about survival improvement and is not different from screening at 6-monht intervals [23, 24]. In studies of HBV patients, 6-month screening resulted in improved survival compared to 12-month screening [25].

## 5. Availability of Effective Therapy

The last 10–15 years have witnessed the advent of newer treatment options for hepatocellular carcinoma, and with it, some measure improvement in outcomes. With early diagnosis, cure is possible in 30% of cases, and in the rest, effective control is achievable. Surgical resection and local ablation are effective in the treatment of an early, localised hepatocellular carcinoma, and achieving 5-year survival of up to 70%. Liver transplant in well-selected patients can bring about a 5-year survival in the order of 80% [26, 27]. Recent data indicates that RFA is comparable to surgical resection for early hepatocellular carcinoma in terms of survival outcomes and has the advantage of being less invasive [28, 29]. Transarterial chemoembolisation is an option proven to prolong survival in cases of nonresectable, nontransplantable cases of nonmetastatic hepatocellular carcinoma.

## 6. Summary

Surveillance of hepatocellular carcinoma is justified in groups at risk of hepatocellular carcinoma. It allows for its detection at earlier stages. This in turn translates to more effective treatment options resulting in improved survival. HCC screening therefore is an important part of the strategy in improving survival in patients with advanced liver disease. Present screening method is that of AFP and ultrasound performed at 6–12-month intervals.
